# Clinical Implications of High-Sensitivity Cardiac Troponin Measurements in Hospitalized Medical Patients

**DOI:** 10.1371/journal.pone.0117162

**Published:** 2015-01-30

**Authors:** Gideon Y. Stein, Danny Alon, Roman Korenfeld, Shmuel Fuchs

**Affiliations:** Department of Internal Medicine “B”, Beilinson Hospital, Rabin Medical Center, Petah Tikva and Sackler Faculty of Medicine, Tel Aviv, Israel; Sickle Cell Unit, JAMAICA

## Abstract

**Background:**

The increased use of high sensitivity cardiac troponins (hs-cTn), have made the diagnosis of non-ST elevation myocardial infarction (MI) challenging, especially in complex medical patients, in whom the clinical presentation of MI is nonspecific and multiple comorbidities as well as non-ischemic acute conditions may account for elevated hs-cTn levels. The aim of this study was to assess the frequency of both elevated hs-cTn levels and dynamic changes in hospitalized patients.

**Methods and Findings:**

We conducted a retrospective study identifying all patients hospitalized in the Internal Medicine Division of Rabin Medical Center, Israel between January 2011 to December 2011, for whom at least one hs-cTn T (hs-cTnT) measurement was obtained. Collected data included patient demographics, acute and chronic diagnosis, hs-cTnT and creatinine levels and date of death. Hs-cTnT levels were obtained in 5,696 admissions and was above the 99th percentile (> = 13 ng/L) in 61.6% of the measurements. A relative change of 50% or higher was observed in 24% of the admissions. Among those with elevated hs-cTnT levels, acute coronary syndromes (ACS) accounted for only 6.1% of acute diagnoses. Maximal hs-cTnT levels above 100 ng/L but not dynamic changes discriminated between ACS and non-ACS conditions (positive and negative predictive values of 12% and 96% respectively). The frequency of elevated hs-cTnT levels was age-dependent and over 75% of patients aged >70 years-old had levels above the 99th percentile. Multivariate analysis identified hs-cTnT levels higher than the 99th percentile, as an independent, strong predictor for 30-day mortality (OR 4.58 [2.8, 7.49], p<0.0001).

**Conclusions:**

Elevated hs-cTnT levels together with dynamic changes are frequent findings among hospitalized patients and in most cases, are not related to the ACS diagnosis. These findings highlight the diagnostic challenge of ACS in this complex population. Further studies are needed in order to optimize the use of hs-cTnT measurements in hospitalized patients.

## Introduction

The 3^rd^ universal definition of myocardial infarction using high-sensitivity troponin (hs-cTn) is broadly applied to rule out acute myocardial infarction (AMI) with very high negative predictive values of 97–100%.[1–3] However, due to the reciprocal relation between sensitivity and specificity of the assay, the positive predictive values for AMI are lower, ranging in selected patient populations between 50% to 84%.[2,3] While in these studies the prevalence of AMI was 17%, it was estimated that in a typical chest pain unit, where the probability of AMI is ≈5%, a larger percentage of patients with elevated hs-cTn levels above the 99th percentile not meeting criteria for AMI, will be evident.[4] Apart from this important Bayesian projective, it is currently well recognized that hs-cTn levels are frequently elevated in various cardiac and non-cardiac clinical conditions unrelated to acute coronary syndromes (ACS) and frequently carry prognostic value. [3,5,6] Additional factors such as age and renal function were also found to affect hs-cTn levels.[7–9] These factors, along with analytical issues, were recently underscored as potential important hurdles in the practical interpretation of hs-cTn measurements, especially in the hospitalized patient population in which frequent cardiac comorbidities are to be anticipated.[10] Indeed, data from the pre- high sensitivity troponin era suggested that troponin levels are elevated in 1 of 4 hospitalized patients, in whom non-ACS causes account for 58% of the cases.[5] Accordingly, the aims of the current study were twofold: 1) to explore the frequency of elevated hs-cTnT and dynamic changes, obtained according to common daily practice, among hospitalized patients with ACS, cardiac and non-cardiac medical conditions, and 2) to assess the impact of hs-cTnT levels and dynamic changes on early mortality.

## Methods

We conducted a retrospective study identifying all patients whose visit to the emergency room led to hospitalization in the Internal Medicine Division, which includes one Geriatric and nine Internal Medicine wards, at The Rabin Medical Center, Israel between Jan 1^ST^ 2011 to December 31^st^ 2011. Collected data included age and gender, ICD-9 codes of acute and chronic diagnosis, hs-cTnT and creatinine blood test values and date of death.

Patients were included if at least one hs-cTnT measurement was obtained.

Hs-cTnT was measured with highly sensitive assay (Troponin T hs Stat; Roche Diagnostics, Indianapolis, IN, USA). According to the manufacturer, the lowest measurable concentration is 5 ng/l, the limit of blank is 3 ng/L, the coefficient of <10% is 13 ng/L and the 99th percentile of a healthy reference population is <13 ng/L. Accordingly, all hs-cTnT, equal or below the 99th percentile are reported by the laboratory as a value of <13 ng/ml. The frequency of a 50% change from the minimal hs-cTnT recorded for that patient during the hospitalization [11] and the frequency of a 50% change from the first measurement of the current admission (base-line) were recorded for patients for which at least two hs-cTnT measurements were obtained during hospitalization.

Hs-cTnT was obtained at the discretion of the treating physician in the Emergency Department. The indication for hs-cTnT level measurement in our hospital includes: primarily evaluation of chest pain, dyspnea or atypical symptoms compatible with a presentation of ACS, or conditions potentially associated with myocardial injury such as pulmonary emboli, sepsis, (peri)myocarditis, hypertensive emergencies and certain arrhythmias.

Demographics, acute and chronic diagnoses and blood tests data were obtained from Rabin Medical Center’s Electronic Medical Record system. Mortality data were acquired from the Rabin Medical Center registry, which is updated monthly from the Israeli Ministry of Internal Affairs registry.

The study was approved by Rabin Medical Center’s institutional ethics committee. Written informed consent was not requested from participants as the clinical data used for this study was anonymized and de-identified prior to analysis.

### Statistical Analysis

To best differentiate between patients with ACS and those with other diagnoses, we performed optimization analysis of maximal hs-cTnT and dynamic changes. Performance was assessed by calculating the maximal average recall, defined as [(% of patients with ACS above the hs-cTnT cutoff level for AMI) + (% of patients with different diagnosis below the cutoff)]/2. A JAVA program was developed, which ran over the maximal hs-cTnT range and the dynamic changes range, calculating precision for each combination of hs-cTnT level and dynamic range cutoff combinations.

Statistical analysis was performed with SPSS software for Windows (SPSS Inc., Chicago, IL, USA); continuous variables are expressed as a mean ± 1 standard deviation (SD) and as a median [interquartile range], when indicated. Categorical variables are presented as percentages. Comparisons between the two groups were performed by Student’s t-test for continuous variables and the chi-square test for comparison of categorical values. Survival was compared between groups using Kaplan-Meier survival analysis and Cox proportional hazards analysis. Backward elimination was used for all variables. All tests of significance were performed using two-tails, with a p value <0.05 considered significant.

## Results

### Patient characteristics

We evaluated 18,830 admissions of 13,029 patients. The final cohort comprised of 4615 (35.4%) patients for whom hs-cTnT was obtained.

Baseline characteristics are detailed in Table 1. Cardiovascular and non-cardiovascular comorbidities were present in 71% and 46% of the patients, respectively. The most frequent acute diagnosis at discharge was non-specific chest pain, followed by heart failure, anemia and chronic obstructive pulmonary disease (COPD) exacerbation. Final diagnosis of ACS accounted for 6.1% of admissions (Table 1).

**Table 1:** Patient Characteristics.

Parameter	Value
Age (average±SD)	71.5±15
Male gender (%)	53.5
Acute diagnosis (%)	
Nonspecific chest pain	24.1
Acute coronary syndrome	2.1
Myocardial infarction	4
Heart failure	23
Anemia	21.9
COPD exacerbation	17.2
Acute renal failure	6.3
Pulmonary emboli	2
Sepsis	2
Comorbidities (%)	
Ischemic heart disease	41.7
Heart failure	24.3
Post CVA	14.1
Diabetes	35.7
Hypertension	56.1
Chronic renal failure	19.4
Atrial Fibrillation	25.2
COPD	16.9
Malignancy	13.7

### Hs-cTnT levels and dynamic changes

**Fig 1:**
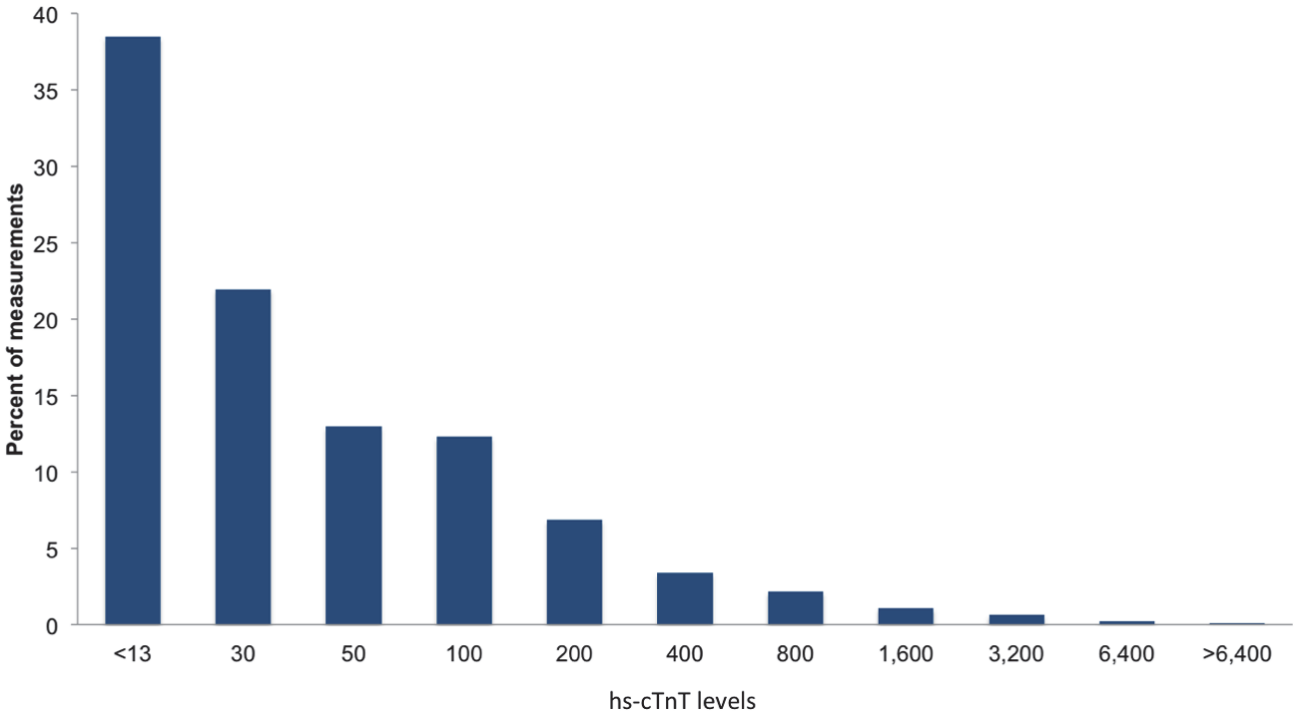
High sensitivity troponin value distribution. Distribution of hs-cTnT levels (expressed as ng/L) among 10,021 measurements. Only 38.4% of the measurements were below the 99th percentile.

**Fig 2:**
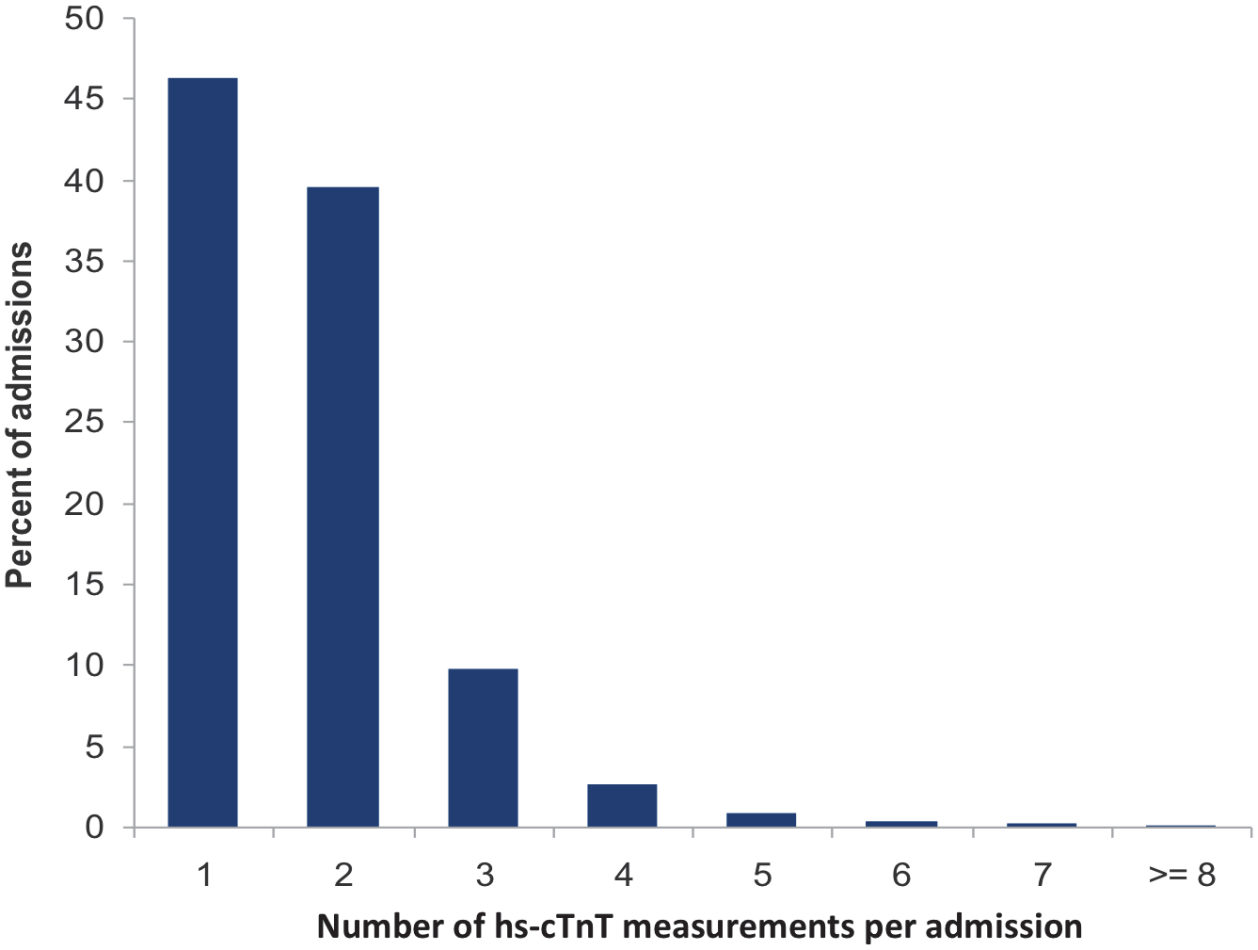
Distribution of high sensitivity troponin measurements per admission. Two or more hs-cTnT measurements were obtained in a little more than half of the hospital admissions in which Hs-cTnT measurements were observed.

Hs-cTnT analysis included 10,021 measurements. Median hs-cTnT levels were 21 ng/L, interquartile range [10, 53] and only 38.4% of the measurements were equal or below the 99th percentile (Fig. 1). A single measurement was obtained in 46% and serial measures in 54% of the admissions (Fig. 2). Among the 3,062 admissions in which more than one hs-cTnT measurement was obtained, a hs-cTnT delta-change of 50% or higher was observed in 24% of the patients. The median time difference between the first and second hs-cTnT measurement (for patients with serial hs-cTnT measurements), was 14.8 hours, interquartile range [8.3–22.7] hours.

Hs-cTnT delta change (maximum minus minimum value) and hs-cTnT delta change from 1^st^ measurement (maximum minus 1^st^ hs-cTnT measurement) were highly correlated (correlation coefficient 0.8, p<0.0001).

**Fig 3:**
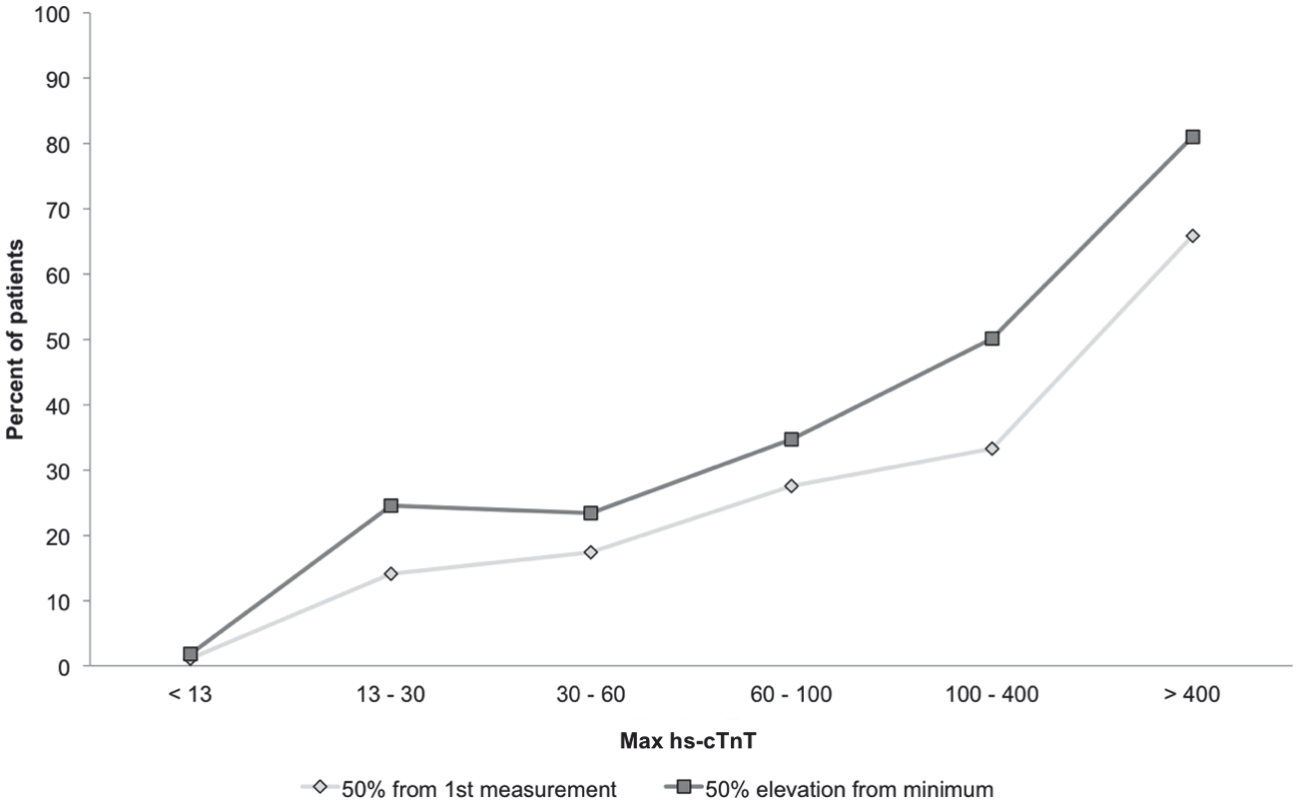
Relative change by maximal high sensitivity troponin levels. The percent of admissions having hs-cTnT dynamics of 50% or more from minimum value or from admission baseline correlates with the maximal hs-cTnT level, p for trend <0.0001 for both.

Hs-cTnT delta change as well as hs-cTnT delta change from 1^st^ measurement significantly correlated with maximum hs-cTnT level per admission (p<0.0001 for all measurements) (Fig. 3).

### Hs-cTnT and patient age

**Fig 4:**
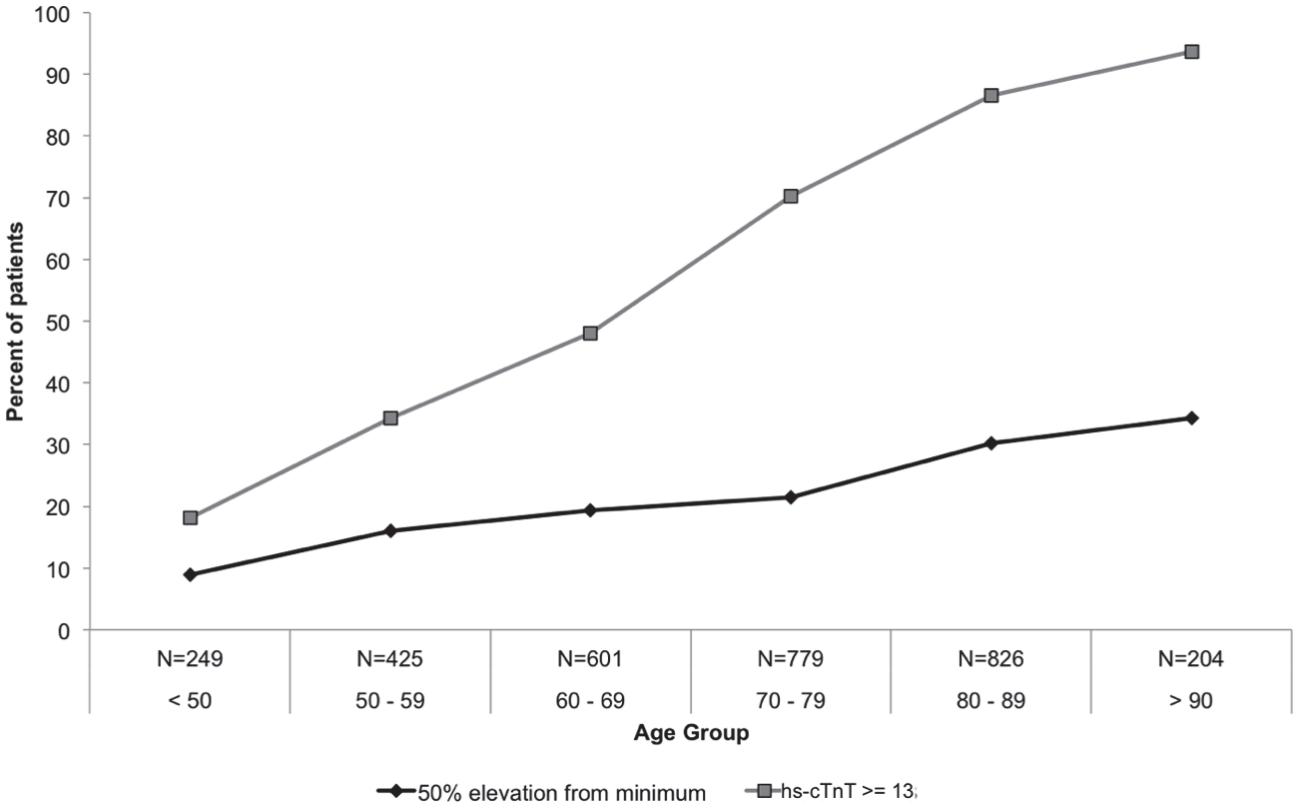
High sensitivity troponin levels and dynamics by age group. The percent of admissions having maximal high sensitivity troponin levels > 99^th^ percentile as well as those with hs-cTnT change of 50% or more correlate with patient age (p for trend < 0.0001 for both).

Hs-cTnT levels showed an increase with age; a maximum hs-cTnT level ≥13 ng/ml was found in 18% of patients < 50, with frequency rising steadily to above 92% among nano-genarians (p<0.0001) (Fig. 4). A similar trend was noted for dynamic changes, whereas a hs-cTnT level change of 50% or higher was noted in 10% of patients <50 and was three fold more frequent among older patients >90 (p<0.0001) (Fig. 4).

### Hs-cTnT and renal function

**Fig 5:**
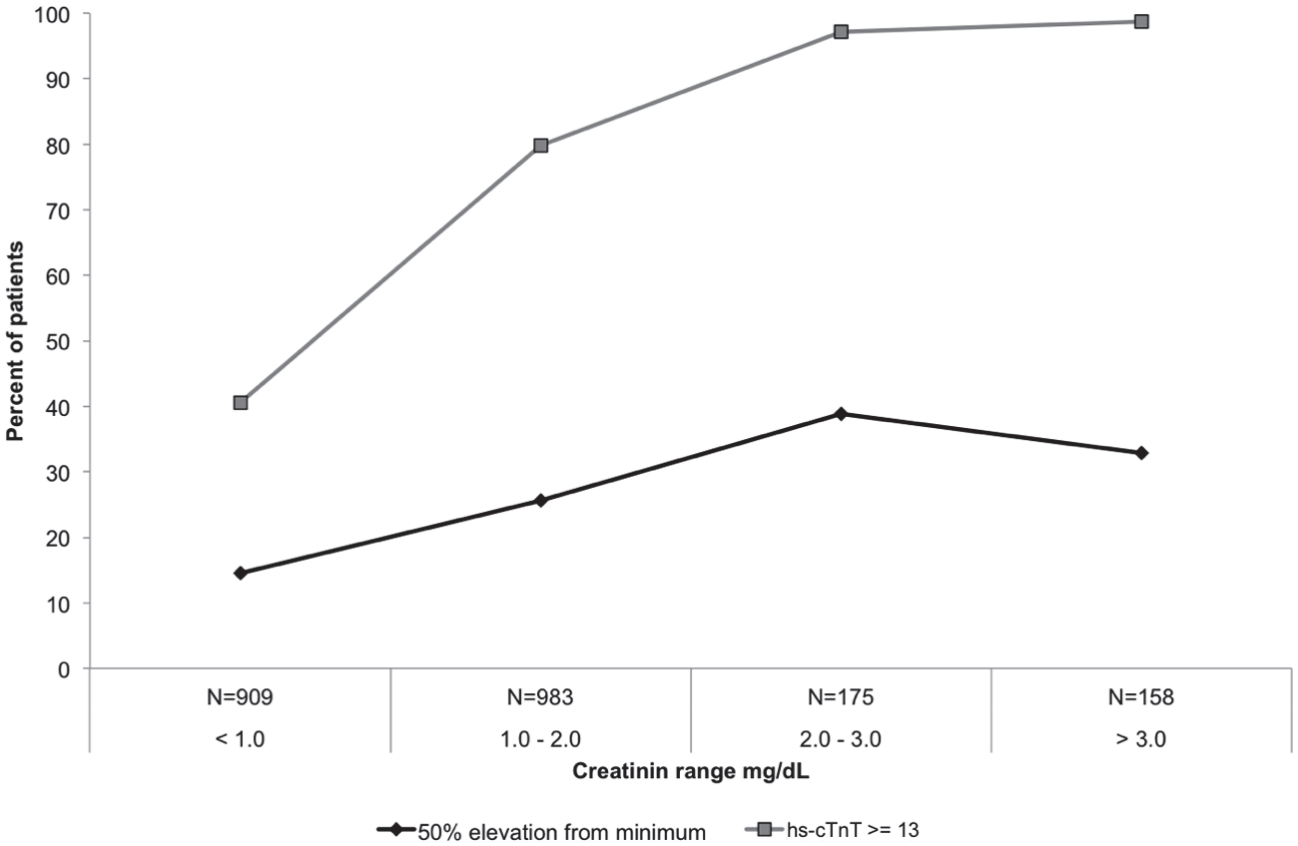
High sensitivity troponin levels and dynamics by creatinine levels. The percent of admissions having maximal hs-cTnT levels > 99^th^ percentile as well as those with hs-cTnT change of 50% or more correlate with patient creatinine levels (p for trend < 0.0001 for both).

Hs-cTnT levels 13 ng/L closely correlated with creatinine levels (Fig. 5). These elevated levels were observed in 41% of patients with creatinine levels below 1 mg/dL and in nearly all patients with creatinine levels > 3 mg/dL (r = 0.153, p<0.0001). Similarly, a two-fold increase (from 16% to 33%) was evident in patients with a dynamic hs-cTnT change of 50% or higher among patients with creatinine levels < 1 mg/dL and > 3 mg/dL, respectively (r = 0.105, p<0.0001) (Fig. 5). Absolute dynamic change of 9 ng/L was also strongly correlated with creatinine levels (r = 0.34, p<0.0001).

### Hs-cTnT and primary discharge diagnosis

**Fig 6:**
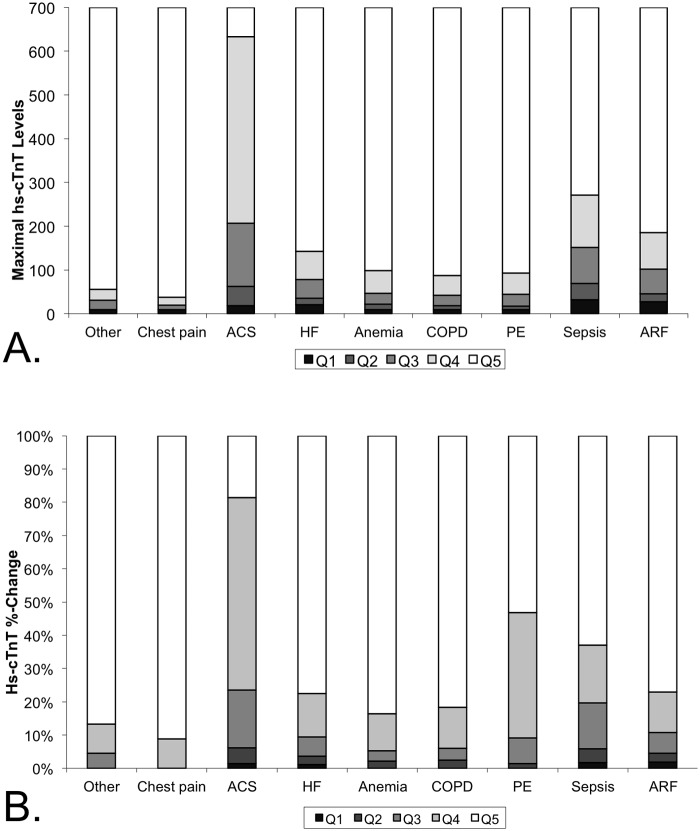
Quintiles of hs-cTnT levels and dynamics by acute diagnosis. Segmentation of high sensitivity troponin levels (A) and dynamic changes (B) to quintiles per discharge diagnosis show that most patients admitted with conditions other than chest pain had high sensitivity troponin levels above the 99^th^ percentile (A) and are likely to have high sensitivity troponin dynamic changes (B).

To analyze the differences between patients with ACS and those with other diagnoses, the maximal hs-cTnT levels and dynamic changes were segmented into quintiles for each discharge diagnosis. Patients with acute diagnosis of ACS/MI and sepsis compared to those with other acute diagnoses, had higher maximal hs-cTnT levels as well as higher dynamic changes (Fig. 6).

**Fig 7:**
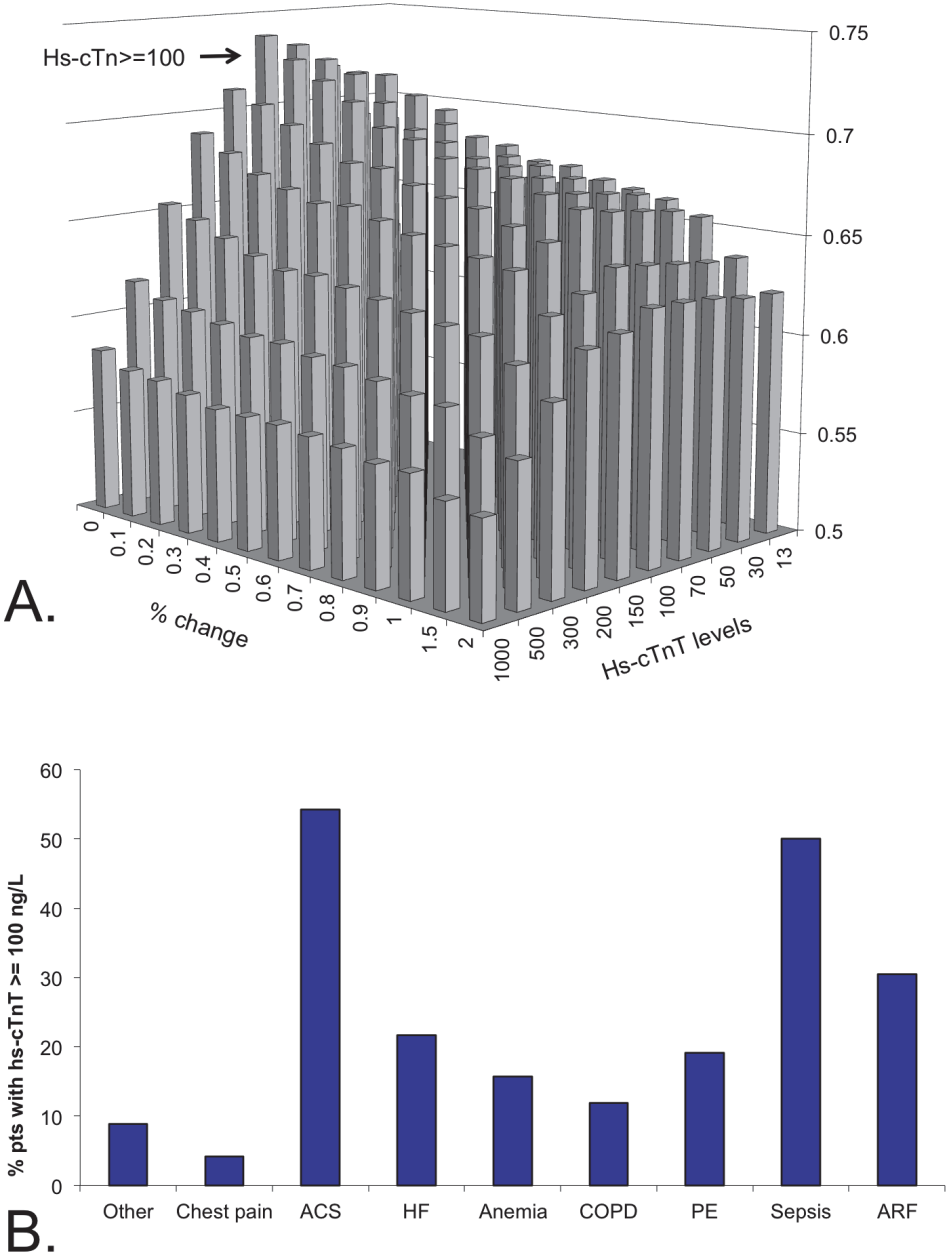
Optimization of maximal high sensitivity troponin and dynamic change to differentiate between ACS and other diagnoses. A graphic presentation of a goal-seeking algorithm assessing different cutoff values of high sensitivity troponin levels and dynamics between high sensitivity levels (A) and the percent of patients with elevated high sensitivity troponin > 100 ng/L (B).

To best differentiate between patients with ACS and those with other diagnoses, we performed a goal-seeking analysis to find optimal cutoff values of maximal hs-cTnT levels and dynamic changes. We found that maximal hs-cTnT levels but not dynamic changes best differentiated between patients with ACS and those with other diagnoses (Fig. 7A). Sixty percent of patients with ACS had maximal hs-cTnT levels ≥ 100 ng/L. while only 12% of patients with other diagnoses had hs-cTnT levels ≥ 100 ng/L. (Fig. 7B). The positive predictive value of the hs-cTnT cutoff of 100 ng/L was 12% and the negative predictive value was 96%.

### Hs-cTnT in young patients without ACS

Patients < 60 years with normal renal function and an acute diagnosis other than ACS, heart failure or COPD accounted for 11.6% of the cohort. Among them, 85% had maximal hs-cTnT levels < 13 ng/L and 86% had hs-cTnT change < 50%.

### Hs-cTnT and 30-day mortality

**Fig 8:**
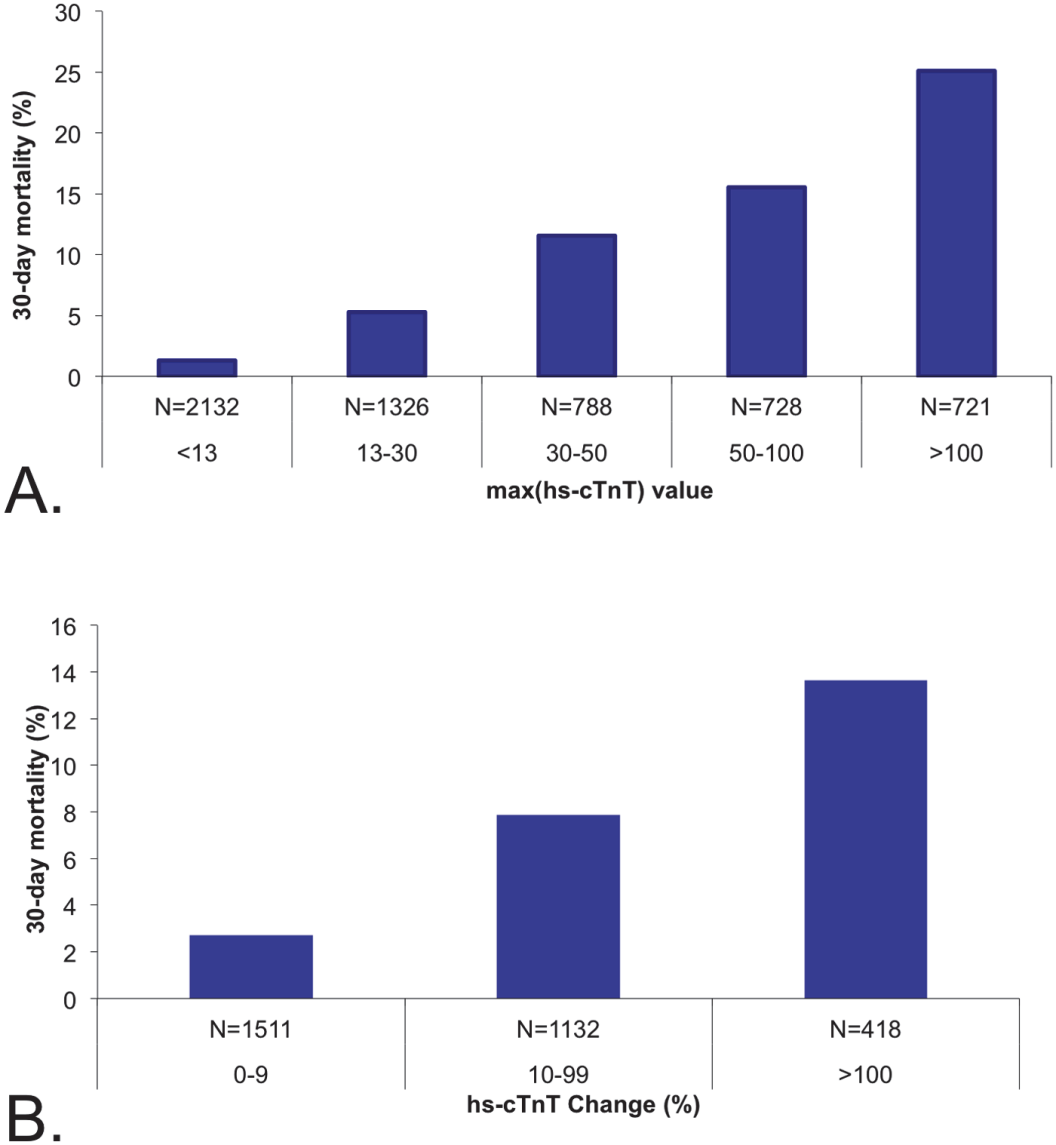
30-day mortality by high sensitivity troponin levels. Univariate analysis showed that maximal high sensitivity troponin levels (A) as well as high sensitivity troponin dynamics correlated with early patient death (p<0.0001 for both).

Elevated hs-cTnT levels were associated with a significant stepwise increase in 30-day mortality rates, ranging between 1.3% among patients with levels <13 ng/L and 25% among those with levels >100 ng/L (p<0.0001) (Fig. 8). The magnitude of dynamic changes in hs-cTnT levels also correlated with 30-day mortality. Mortality rates for patients without significant changes (defined as <10%) had 2.7% mortality rates whereas for those who had an increase ≥100% had 13.6% 30-day mortality, (p<0.0001) (Fig. 8B).

Multivariate analysis identified hs-cTnT levels ≥13 ng/L, but not dynamic change or absolute changes, as a strong predictor for a 30-day mortality (OR 4.58 [2.8, 7.49], p<0.0001) (Table 2). Importantly, the prognostic value of elevated hs-cTnT was similar after excluding patients with a major diagnosis of sepsis (OR 4.88 [2.95, 7.82], p<0.0001.

**Table 2:** Multivariate analysis for 30-day survival.

Parameter	OR (95% CI)	p
Age	1.03 (1.02, 1.04)	<0.0001
Creatinine	1.17 (1.1, 1.26)	<0.0001
Chest Pain	0.18 (0.1, 0.32)	<0.0001
Sepsis	11.17 (7.27, 17.17)	<0.0001
ARF	1.66 (1.18, 2.33)	0.004
ACS	0.46 (0.27, 0.79)	0,005
hs-cTnT>0.013	4.58 (2.8, 7.49)	<0.0001

ARF denotes acute renal failure; ACS denotes acute coronary syndrome. hs-cTnT denotes high sensitivity troponin

## Discussion

In this study we analyzed hs-cTnT measurements in a large cohort of hospitalized medical patients. We report four major findings: 1) elevated hs-cTnT levels above 99^th^ percentile are found in > 60% of all patients, 2) in the vast majority of patients, elevated hs-cTnT levels are due to *non*-ACS and *non*-acute cardiac conditions, 3) dynamic changes in hs-cTnT are similarly common among patients with and without ACS and 4) elevated hs-cTnT above the 99^th^ is associated with more than a 4-fold increase in 30-day mortality.

The percentage of patients with elevated hs-cTn levels above the 99th percentile varies and is considerably affected by the patient population being studied.[12] In the Atherosclerosis Risk in Community study, 13% of men and 3% of women had a hs-cTn level above the 99th percentile.[13] Analysis from the Cardiovascular Health Study of community—based population >65 years without heart failure showed that 16.6% of the participants had hs-cTn levels close or higher than the 99th percentile. [6] A recent analysis of those studies suggests that a potentially more accurate approach should include age- and gender-specific 99^th^ percentile values.[14] Such strategy may allow avoiding over diagnosis of myocardial injury among elderly men and under diagnosis among young women. The frequency of elevated hs-cTn above the cutoff for myocardial injury among patients admitted to the emergency room, in whom ACS and other potential conditions known to be associated with elevated hs-cTn were excluded, was low among patients > 65 years but reached 27% among patients > 65 years.[12] In practice, however, hs-cTn is obtained in a wide range of patients being admitted and among this population non-hs-cTn was shown to be elevated in 25% of patients.[5] Our study expands these observations and underscores the high frequency of elevated hs-cTnT among a large, unselected, real world patient-population without ACS. These high rates probably reflect the increased age and presence of multiple cardiac and non-cardiac comorbidities in which hs-cTn is frequently elevated. Support for this observation arises from our findings regarding the prevalence of elevated hs-cTn levels among subpopulations. The frequency of elevated hs-cTnT among patients < 60 years without ACS, renal failure or COPD was 15%, while the prevalence in patients > >70 and 80 years was 70% and 80%, respectively. Similarly high rates were observed among patients with renal failure strongly correlated to creatinine levels. Hence, an important potential practical implication of our observations is an expectation of elevated hs-cTn levels among a high percentage of admitted patients, especially in the elderly and those with renal failure.

An interesting finding of the current study is that nearly 94% of the patients with elevated hs-cTnT above the 99^th^ percentile had a final non-ACS diagnosis. It is thus conceivable that although hs-cTn levels may facilitate the identification of more patients with AMI, its utilization will result in many- fold increase in the number of patients with non-ACS conditions associated with minor myocardial injury.[4,11,15,16] Several hs-cTn cutoffs and dynamic changes were suggested to distinct between patients with and without ACS related myocardial injury.[11,17] In the current study we found that among hospitalized patients with elevated baseline hs-cTnT levels, the dynamic change of 50% was similarly common among those with ACS and other acute diagnoses. Nevertheless, as our study was not designed to assess specific time-dependent changes in hs-cTnT levels, we cannot rule-out whether specific time-dependent dynamic changes would better distinct between ischemic compared to other myocardial injuries.

In the current study we observed a dynamic change of ≥50% in hs-cTnT levels in 24% of those who had serial measurements. It is conceivable that at least some of these patients may have experienced type II AMI. The true incidence of type II AMI is currently unknown. We previously reported, using a large national survey data base, a prevalence of 4.5% and 7% among those diagnosed with any AMI and NSTEMI, respectively. [18] Other studies revealed a varied frequencies, range between 1.6–62%. [19] This heterogeneity may reflect different definitions of significant hs-cTn change as well as the complexity of clinical interpretation of ambiguous, non-specific symptoms and inconsistent utilization of supportive cardiac imaging. It is conceivable, therefore, that part of our cohort with elevated dynamic hs-cTn changes experience type II AMI.

The association between hs-cTn levels and outcomes is well established in multiple cardiac and non-cardiac conditions as well as among patients admitted to non-cardiac intensive care units. [5,20–24] In the current study we found that elevated hs-cTnT levels above the 99^th^ percentile is a strong predictor for 30-day mortality, second only to sepsis. As 94% of our cohort had a non-ACS diagnosis, the strong association between hs-cTnT levels and increased mortality reflect its impact in a large non-ACS hospitalized population. A previous follow-up report of discharged patients showed that elevated troponin levels were associated with higher 1-year mortality among non-ACS compared to ACS patients.[5] Whether elevated hs-cTn among non-ACS patients is a marker of more severe underlying comorbidities is currently unknown.

### Limitations

The current study carries inherent limitations of a retrospective analysis. First and foremost was the lack of data in relation to the indication for hs-cTnT measurements. Hs-cTnT was overall measured in 30% of admissions, thus the results may not represent an unselected hospitalized cohort. Furthermore, data regarding structural heart disease, which may affect hs-cTnT levels is lacking. However, considering the high prevalence of other comorbidities associated with elevated hs-cTnT levels among our cohort, including age, heart failure, diabetes, ischemic heart disease, hypertension and renal failure it is conceivable that structural changes such as left ventricular hypertrophy and systolic dysfunction were highly prevalent among our cohort. Finally, this observational study is also lacking systematic assessment of myocardial injury using non-invasive imaging modalities. Such data could better improve distinction between ACS and non-ACS related myocardial injuries.

### Conclusions

Elevated levels and significant dynamics of hs-cTnT levels are frequent findings among hospitalized patients, in whom the vast majority have non-ACS acute diagnosis. In this cohort, ruling-out ACS is challenging and studies aimed to optimize utilization of hs-cTnT measurements to identify ACS in this complex population are warranted.
